# Sine‐wave electrical stimulation initiates a voltage‐gated potassium channel‐dependent soft tissue response characterized by induction of hemocyte recruitment and collagen deposition

**DOI:** 10.14814/phy2.12832

**Published:** 2016-06-22

**Authors:** Brandon M. Franklin, Eleni Maroudas, Jeffrey L. Osborn

**Affiliations:** ^1^ Department of BiologyUniversity of KentuckyLexingtonKentucky

**Keywords:** Electrical stimulation, hemocyte, macrophage, voltage‐gated potassium channels

## Abstract

Soft tissue repair is a complex process that requires specific communication between multiple cell types to orchestrate effective restoration of physiological functions. Macrophages play a critical role in this wound healing process beginning at the onset of tissue injury. Understanding the signaling mechanisms involved in macrophage recruitment to the wound site is an essential step for developing more effective clinical therapies. Macrophages are known to respond to electrical fields, but the underlying cellular mechanisms mediating this response is unknown. This study demonstrated that low‐amplitude sine‐wave electrical stimulation (ES) initiates a soft tissue response in the absence of injury in *Procambarus clarkii*. This cellular response was characterized by recruitment of macrophage‐like hemocytes to the stimulation site indicated by increased hemocyte density at the site. ES also increased tissue collagen deposition compared to sham treatment (*P* < 0.05). Voltage‐gated potassium (*K*_V_) channel inhibition with either 4‐aminopyridine or astemizole decreased both hemocyte recruitment and collagen deposition compared to saline infusion (*P* < 0.05), whereas inhibition of calcium‐permeable channels with ruthenium red did not affect either response to ES. Thus, macrophage‐like hemocytes in *P. clarkii* elicit a wound‐like response to exogenous ES and this is accompanied by collagen deposition. This response is mediated by *K*_V_ channels but independent of Ca^2+^ channels. We propose a significant role for *K*_V_ channels that extends beyond facilitating Ca^2+^ transport via regulation of cellular membrane potentials during ES of soft tissue.

## Introduction

Repair of soft tissue damage is a critical need of living organisms that involves basic restoration of anatomical structures and functions to damaged tissue. This is a delicate process and its execution is not always optimal, as either excessive healing (e.g., fibrosis and adhesions) or inadequate healing (e.g., chronic wounds and ulcers) can lead to diminished or lacking restoration of function (Lazarus et al. 1994; Diegelmann and Evans 2004). An essential mediator of the wound repair process is the infiltration of the wound region with macrophages (Clark 1988). Disruption of normal macrophage activity in wound healing can contribute to decreased inflammatory cytokines, neutrophil removal, angiogenesis, fibroblast proliferation and collagen deposition (Gardner et al. 1999; Mirza et al. 2009; Koh and DiPietro 2011; Clark 1988). Conversely, activation or introduction of macrophages at the site of the wound accelerates wound healing in experimental models of impaired wound healing (Danon et al. 1989; Chen et al. 2008). Wound induced electrical fields (EFs) are an intrinsic property of damaged tissues that are vital to the wound healing process across different species (Chiang et al. 1991; Jenkins et al. 1996; Reid et al. 2005; Wang and Zhao 2010; Messerli and Graham 2011). Also, macrophages are sensitive to electrical fields and this property may be somewhat responsible for the positive effects of electrical stimulation (ES) on wound healing and repair of neural tissue (Moriarty and Borgens 1998; Cho et al. 2000).

These findings suggest that wound induced EFs direct macrophage migration to the wound site but few studies identify the mechanisms responsible for facilitating this action. There are long‐standing hypotheses that endogenous ionic currents act to control cell dynamics in development, wound healing and regeneration (Jaffe and Nuccitelli 1977; Borgens et al. 1979; Jaffe 1981; Özkucur et al. 2010). However, the mechanisms utilized by cells to detect the EF and translate it into a discernable message to drive specific cell behaviors, such as migration, proliferation, and differentiation, are not well understood. A better understanding of how cells are able to sense EFs and react to them is vital to understanding the global physiology involved in tissue repair. Ion channel signaling provides a reasonable cellular response for mediating these effects based on their documented involvement in cell proliferation, migration, and differentiation (Lang et al. 2005; Prevarskaya et al. 2007; Schwab et al. 2012). Specifically, voltage‐gated and calcium‐sensitive potassium channels are involved in regulating a variety of macrophage functions including activation, migration and cytokine secretion (Gallin 1984; Mackenzie et al. 2003; Dong et al. 2013).

This study investigated this phenomenon using an invertebrate homolog of macrophages, crustacean hemocytes, by measuring how they respond to exogenous ES and how this response is mediated by both potassium and calcium channel signaling. The response to ES was assessed using basic histological techniques and pharmacological antagonists were used to assess the role of K^+^ and Ca^2+^ channels in hemocyte recruitment during ES.

## Methods

### Animal preparation

**Figure 1:**
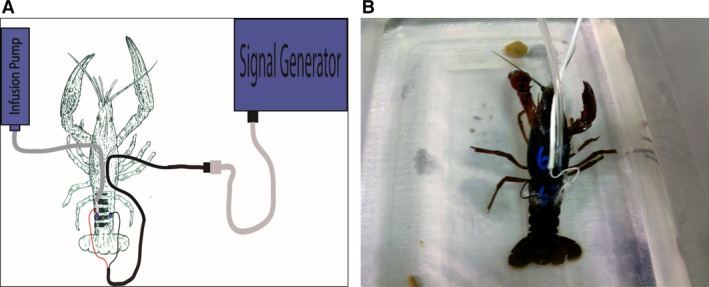
(A) Schematic drawing of the experimental preparation. Electric stimulation is supplied by the signal generator and pharmacological inhibitors are delivered via a syringe infusion pump. (B) Photographic representation of an instrumented crayfish.

Adult *Procambarus clarkii* ranging from 3 to 5 inches in length were procured from Atchafalay Biological in Raceland, LA and allowed to acclimate to the laboratory aquatic living environment for at least 2 weeks. Animals that were in the process of molting or had recently molted were excluded from the study. Animals selected for this study were anesthetized in ice water for 30 min before being instrumented for the experiment. A small hole (2 mm × 2 mm) was made in the third segment of the dorsal tail carapace. Stainless steel electrode tips (4 mm × 2 mm) were implanted between the carapace and the tail muscle surface with the cathode on the left and the anode on the right. For experiments requiring infusion of pharmacological agents or vehicle, polyethylene tubing connected to an infusion pump was inserted into the same hole as the cathode (Fig. 1). Carapace opening closure and securing of the instrumentation was conducted using fast drying cyanoacrylate (commercial superglue) applied to the openings. Animals then were placed in individual water‐filled enclosures and allowed 2‐4 recovery days before beginning the experiment.

### Treatment protocol

Following the 4‐day recovery period, drug or vehicle infusion was initiated. Acclimation for 24 h was allowed to ensure total blockade of targeted ion channels before initiating electrical stimulation. Tail muscles were intermittently stimulated with sine‐wave impulses at 450 mV and 2 Hz (50 msec on to 450 msec off) continuously for 4 days. Immediately following the 4‐day stimulation period, animals were anesthetized in ice water and killed by decapitation. Evans Blue dye was injected (10 *μ*L) through the electrode opening of the carapace to mark the site of electrode implantation. The tail muscle then was exposed and a ~20 mm cross section containing the tissue subjected to ES was excised. Tail muscle was fixed by submersion in 4% paraformaldehyde for 12–18 h. Tissues were rinsed in PBS and cryoprotected in 15% sucrose for four hours followed by 30% sucrose overnight, frozen and stored at −20°C. Tissues were embedded in OTC compound and cut into 15 micron tissue sections by cryostat sectioning and mounted onto gelatin‐coated slides. Tissue sections were stained with hematoxylin and eosin (H&E), Masson's trichrome, or picrosirius red according to standard protocols.

### Ion channel inhibition

To examine the role of potassium and calcium channels, pharmacological antagonists were solubilized in standard crayfish saline and infused at 1.25 *μ*L/min before and during the ES. To block voltage‐gated potassium (*K*_V_) currents, 4‐Aminopyridine (4‐AP) was infused (1.25 *μ*L/min) at a concentration of 10 μmol/L and Astemizole (AZ) was infused (1.25 *μ*L/min) at a concentration of 5 μmol/L. Calcium (Ca²⁺) signaling was investigated by infusion of ruthenium red (RR) at 10 μmol/L (Hirano et al. 1998; Taglialatela et al. 1998; Nattel et al. 2000; Clapham et al. 2001; Wulff et al. 2009). Crayfish saline was infused as vehicle control. Infusion commenced 24 h prior to initiation of the experiment to ensure the entire tail muscle was thoroughly bathed in each compound preceding ES onset. When infusion was initiated, crayfish were observed out of their environments for 20 min to ensure there was no leakage of the infusate around the surgical sites or other areas. In total, there were five treatment groups: Sham electrode implantation + saline infusion (Sham/Saline), ES electrodes + saline infusion (Stim/Saline), ES electrodes + 4‐AP infusion (Stim/4‐AP), ES electrodes + AZ infusion (Stim/AZ), and ES electrodes + RR infusion (Stim/RR). All groups were *n* = 6.

### Analysis of hemocyte activation and collagen deposition

To measure the level of hemocyte activation, histological H & E stained sections were examined. Hemocytes within 500 *μ*m of the electrode implantation site were morphologically identified and assigned to one of three groups: granulocytes, semigranulocytes, and hyaline cells. Hyaline hemocytes are characterized by their relatively small size, elongated oval shape, a centralized nucleus, high nuclear/cytoplasmic ratio, and an absence of cytoplasmic granules. Granulocytes are larger with an eccentric, oblong nucleus, lower nuclear/cytoplasmic ratio, and an abundance of eosinophilic granules in the cytoplasm. Semigranulocytes are similar in size and nuclear/cytoplasmic ratio to granulocytes with an eccentric, spherical nucleus, and a reduced number of eosinophilic hemocytes in the cytoplasm (Lanz et al. 1993; Parrinello et al. 2015). Each group then was normalized to tissue section area. Values are expressed as hemocytes per 10,000 *μ*m^2^. Collagen deposition was assayed both qualitatively and semiquantitatively. Qualitative evaluation was achieved with Masson's trichrome staining producing blue collagen, red cytoplasm/muscle fiber, and black nuclei. Semiquantitative assessment was achieved by measuring the percentage of tissue area, within 500 *μ*m (a total tissue area of 0.5 mm^2^) of electrode implantation, staining positive for collagen by polarized light imaging of picrosirius red stained tissue sections.

### Statistical analysis

Statistical differences between or among groups were determined using Student's *t*‐test or one‐way ANOVA. Post hoc analysis was conducted using a Student–Newman–Keuls (SNK) method to determine statistical differences between mean values for each treatment and the control group. All data are presented as mean ± standard deviation. The 0.05 level of probability was utilized as the criterion for significance in all datasets.

## Results

### Intermittent sine‐wave ES elicits hemocyte activation and collagen deposition in crayfish tail muscle

**Figure 2:**
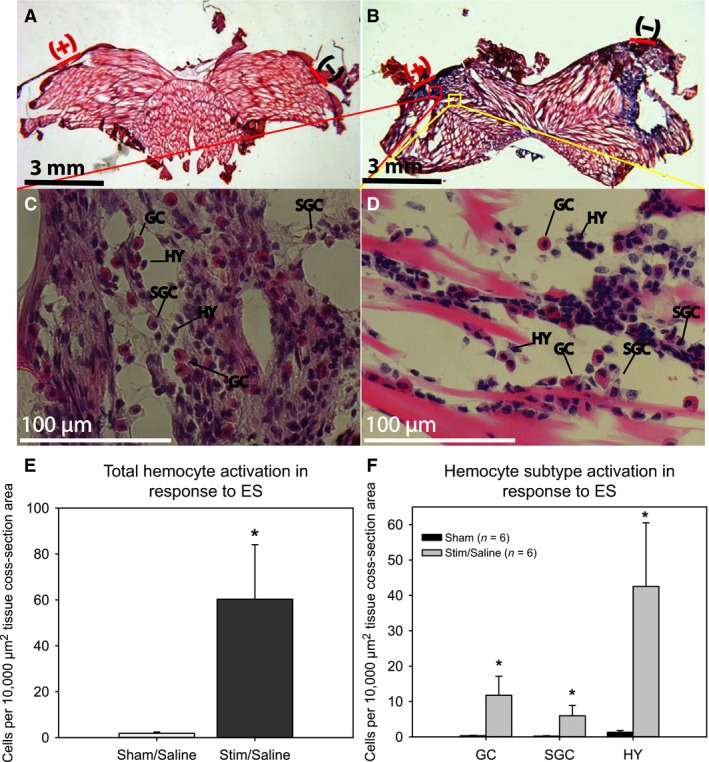
Hematoxylin and Eosin stained tissue sections of sham‐treated (A) and stimulated (B) tail muscle (electrode placement depicted by red dash). An aggregation of hemocytes is observed directly beneath the site of electrode implantation in stimulated (*n *=* *6) but not sham treated (*n *=* *6) animals. Higher magnification of the subject depicted in panels (C & D) show this hemocyte aggregation in detail (GC, granulocyte; SGC, semigranulocyte; HY, Hyaline hemocyte). Hemocyte density near the electrode site was used as a measure of hemocyte activation. (E) Hemocyte density was increased in ES animals (*n *=* *6) compared to sham treated (*n *=* *6) animals. (F) This response was observed in all hemocyte subtypes. (* = *P *<* *0.05 compared to sham/saline; student's *t*‐test).

Following continuous ES over a 4‐day period, the tissue area directly under the site of electrode implantation exhibited an aggregation of hemocytes analogous to the first stage of the wound healing response as described in both penaeid shrimp and freshwater crayfish (Fontaine and Lightner 1973; Fontaine 1975). This hemocyte aggregation was not observed in sham‐treated animals without ES (Fig. 2). Total hemocyte density in animals exposed to exogenous ES was increased (60.29 ± 23.73 hemocytes/10,000 *μ*m^2^) compared to sham‐treated animals (1.87 ± 0.59 hemocytes/10,000 *μ*m^2^, *P* < 0.05), but this effect was not specific to one hemocyte subtype population (Fig. 2).

**Figure 3:**
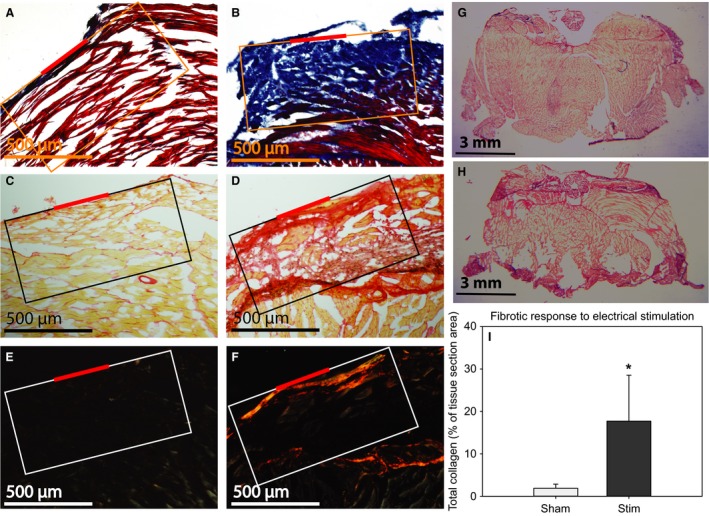
Tissue sections were stained with Masson's trichrome stain (A & B), picrosirus red (PR) under bright‐field (C & D) and PR under polarized light (E & F). Electrically stimulated animals (B, D & F; *n *=* *6) exhibited increased collagen staining compared to sham‐treated animals (A, C & E; *n *=* *6). A less enlarged micrograph of PR stained sham treated (G) and stimulated (H) tail muscle sections depict the overall morphology of the effect. (I) Total collagen was quantified by optical density of PR stained tissue sections imaged under polarized light. Values represent the percentage of a 0.5 mm^2^ area (white box) that stained positive for collagen. (* = *P *<* *0.05 compared to sham/saline; student's *t*‐test).

**Figure 4:**
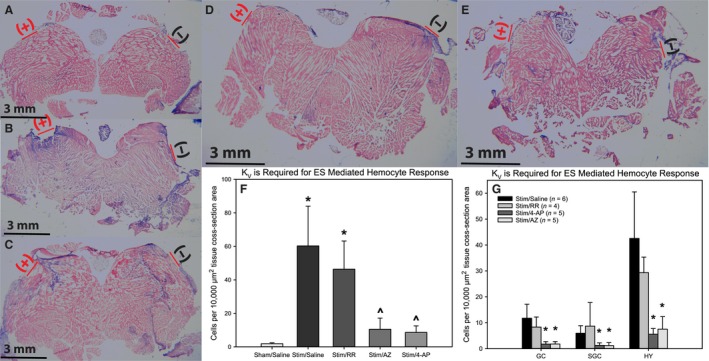
Hematoxylin and Eosin stained tissue sections of sham‐treated/saline infused (A), stimulated/saline infused (B), stimulated/RR infused (C), stimulated/4‐AP infused (D) and stimulated/AZ infused tail muscle (E; electrode placement depicted by red dash). (F) The hemocyte response was attenuated with blockade of *K*_V_11.1 with either astemizole or 4‐aminopyridine. Blockade of TRP Ca^2+^ channels with ruthenium red did not significantly alter the hemocyte response. (G) The influence of *K*_V_ channel blockade is not restricted to any of the hemocyte subtypes. (* = *P *<* *0.05 compared to sham/saline; one‐way ANOVA with SNK post hoc).

Another defining characteristic of the crayfish wound response is the deposition of collagen and subsequent tissue fibrosis (Fontaine and Lightner 1973; Fontaine 1975). Separate histological techniques (Masson's trichrome and picrosirius red staining) were employed to assay for collagen deposition and scarring. Masson's trichrome stain identifies tissue fibrosis and collagen deposition by red cytoplasm, black nuclei, and blue collagen fibers. Sham‐treated animals had minimal collagen deposition adjacent to the electrode implantation site (Fig. 3A). In contrast, animals exposed to exogenous ES exhibited significant collagen deposition directly under the site of electrode implantation (Fig. 3B). These tissues were also stained with picrosirius red and imaged under both bright field (yellow cytoplasm and red collagen) for qualitative assessment and polarized light (yellow‐orange birefringence for thick collagen fibers and green birefringence for thin collagen fibers) for a semiquantitative measurement of total collagen in the tissue adjacent to the site of electrode implantation. Figure 3C and D depict representative sham‐treated (C) and ES (D) animals. Significant collagen deposition is observed in ES animals but not in sham‐treated animals. Using the picrosirius red images taken under polarized light, the percent area of fibrosis was measured. Exogenously stimulated animals exhibited fibrosis in a higher percentage (16.35 ± 5.20%) of tissue adjacent to the electrode implantation site compared with sham‐treated animals (1.47 ± 1.03%, Fig. 4; *P* < 0.05).

### ES mediated hemocyte activation is dependent on *K*_V_ channels

**Figure 5:**
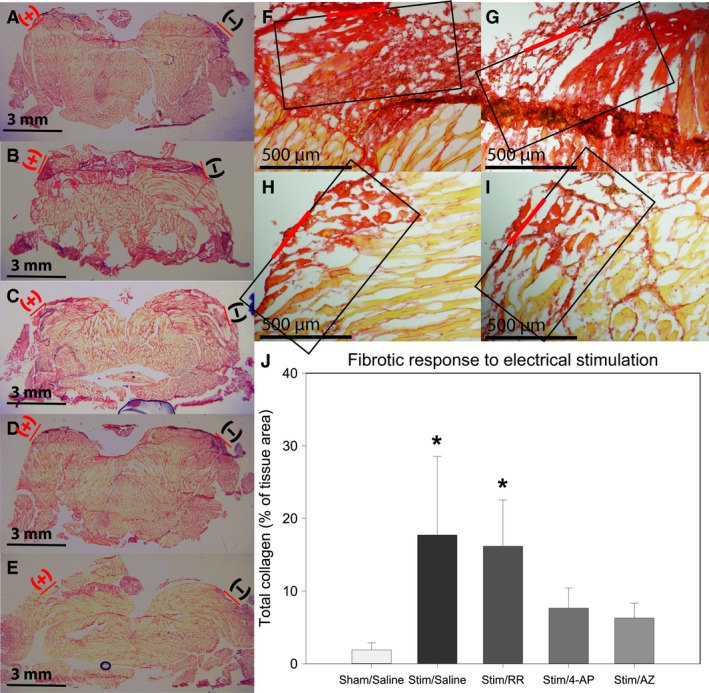
Picrosirus red stained tissue sections of sham‐treated/saline infused (A), stimulated/saline infused (B & F), stimulated/RR infused (C & G), stimulated/4‐AP infused (D & H) and stimulated/AZ infused tail muscle (E & I; electrode placement depicted by red dash). Collagen deposition was reduced by potassium channel blockade by both 4‐aminopyridine (H) and astemizole (I) but was not affected by Ca^2+^ channel blockade with ruthenium red (G) compared to sham‐treated animals (F). (J) Total collagen (% of 0.5 mm^2^ tissue section area; black box) was significantly reduced with blockade of *K*_V_11.1 with either astemizole or 4‐aminopyridine but not by blockade of TRP Ca^2+^ channels via infusion of ruthenium red. (* = *P *<* *0.05 compared to sham/saline; one‐way ANOVA with SNK post hoc).

To assess the role of voltage‐dependent potassium currents in mediating the response to ES, pharmacological modulators were infused continuously (1.25 *μ*L/min) from 24 h before initiation of ES to the end of the experiment. Blockade of *K*_V_ channels with either 4‐AP (10.53 ± 6.65 hemocytes/10,000 *μ*m^2^) or astemizole (8.64 ± 3.89 hemocytes/10,000 *μ*m^2^) decreased the total hemocyte response to ES when compared with saline infusion (60.29 ± 23.73 hemocytes/10,000 *μ*m^2^, *P* < 0.05). This effect was not limited to any specific hemocyte subtype population (Fig. 4). Collagen deposition was also attenuated by either 4‐AP (3.71 ± 0.86%) or astemizole (4.27 ± 1.87%) when compared with saline‐infused animals (16.35 ± 5.20%, Fig. 5, *P* < 0.05).

### Ca^2+^ channels are not required for the ES mediated response

Blockade of Ca^2+^ signaling with ruthenium red (RR) did not affect the hemocyte response to ES (46.38 ± 16.81 hemocytes/10,000 *μ*m^2^) compared to saline‐infused animals (60.29 ± 23.73 hemocytes/10,000 *μ*m^2^, *P* > 0.05; Fig. 4). Collagen deposition also was not changed by RR (11.68 ± 3.84%) when compared with saline‐infused animals (16.35 ± 5.20%, *P* > 0.05; Fig. 5).

## Discussion

The tissue response to low‐amplitude, low‐frequency sine‐wave ES in the tail muscle of adult *P. clarkii* was characterized and compared to documented crustacean wounding responses (Fontaine and Lightner 1973; Fontaine 1975). The response of crayfish hemocytes was of particular interest considering the distinct similarities hemocytes share with macrophages and the documented role of macrophages in vertebrate wound healing (Danon et al. 1989; Hose et al. 1990; Moriarty and Borgens 1998; Söderhäll et al. 2003; Chen et al. 2008; Koh and DiPietro 2011). Second, the study identified *K*_V_ channels as one of the molecular determinants responsible for interpreting the electrical signal into a discernable message to direct cell activity.

Several studies have indicated that wound healing is dependent on a wound‐induced electrical field and that this electrical field can be modulated by exogenous ES (Chiang et al. 1991; Jenkins et al. 1996; Wang and Zhao 2010; Messerli and Graham 2011). This study demonstrated that exogenous ES is sufficient to elicit a soft tissue response in *P. clarkii* tail muscle in the absence of soft tissue injury characterized by hemocyte accumulation and collagen deposition. Hemocyte/macrophage populations have been indicated as essential and early participants in the wound healing process. They play important roles in matrix degradation at the wound site, phagocytosis to remove debris and cytokine secretion to attract other important cell types (Danon et al. 1989; Montagnani et al. 2001; Brancato and Albina 2011; Koh and DiPietro 2011; Clark 1988). In this study, an aggregation of hemocytes was observed in response to ES indicating that this exogenous electrical field stimulates hemocyte recruitment as would happen early on in the wound response. This is not surprising, as in vitro and in vivo studies have shown that macrophages respond to ES. Whether or not ES is directly acting on hemocytes or influencing the action of other cells that cause hemocyte infiltration is unclear but in vitro studies have established a relationship between macrophages and EFs. (Orida and Feldman 1982; Cho et al. 2000; Hoare et al. 2016)

Another typical and consistent characteristic of a wound response is the deposition of collagen (Clark 1988). As shown in Figure 3, the application of ES led to significant collagen deposition. Previously characterized models of crustacean wound responses in penaeid shrimp and *P. clarkii* describe a similar histological response to what has been described in these data (Fontaine and Lightner 1973; Fontaine 1975). These results indicate that low‐amplitude, low‐frequency sine‐wave ES of the crayfish tail muscle brings about some of the typical characteristics of crayfish wound healing in the absence of tissue insult. This provides a basis and rationale for the in vivo study of the molecular mechanisms involved in wound‐induced EF mediated repair processes.

Considering the documented role of potassium channels in both wound healing and macrophage activity, it was reasonable to suspect that they had a role in interpreting the electrical signal to elicit the results seen in this study (Gallin 1984; Blunck et al. 2001; Shin et al. 2002; Anděrová et al. 2004; Kan et al. 2016). In other studies, *K*_V_ channels have been shown to regulate proliferation in multiple tumor cell lines, with strong evidence specifically indicating *K*_V_10.1 and *K*_V_11.1 (EAG and hEAG) channels (Bianchi et al. 1998; Conti 2004). Multiple cell types have demonstrated a dependence on K^+^ channel signaling for proper direction of cell migration (Schwab and Oberleithner 1995; Da Silva‐Santos et al. 2002; Dal‐Secco et al. 2008; Jin et al. 2008; Silver et al. 2015). Recently, a role for K^+^ channel signaling has been demonstrated in regulating both the proliferation of macrophages and their ability to recruit the macrophage precursor Ly6C monocytes (Zhang et al. 2015). The fact that collagen synthesis and deposition is reduced is not surprising, considering that previous studies have found that macrophages stimulate collagen synthesis and scar formation and that specific ablation of macrophage populations before wounding results in reduced collagen deposition (Hunt et al. 1984; Portera et al. 1997; Mirza et al. 2009).

The data fall short of revealing the exact mechanism of *K*_V_ channels’ involvement in this response and *K*_V_ blockade does not completely inhibit the response. ES has been shown to manipulate multiple cellular behaviors including migration, proliferation and cytokine production and it could be that *K*_V_ are only involved in some of these processes. (Fitzsimmons et al. 1992; Li and Kolega 2002; Wang et al. 2003; Kim et al. 2009; Zhao 2009) Further research is needed to understand if *K*_V_ impacts hemocyte proliferation, migration, cytokine secretion, or some other mechanism in the context of wound healing. This study has shown that hemocyte infiltration can be induced via ES and that this model of ES induced hemocyte infiltration also produced collagen deposition. Although, when hemocyte infiltration is blocked, ES alone is not sufficient to induce collagen deposition.

Ca^2+^ permeable channels have been shown to be critical regulators of cell function and are sensitive to potassium channel signaling (Lallet‐Daher et al. 2009; Billeter et al. 2014; Schilling et al. 2014). The classic Ca^2+^ channel inhibitor ruthenium red had no effect on either hemocyte activation or collagen deposition (Figs. 4 and 5). This indicates that Ca^2+^ channel signaling is not a required mediator of the response to ES, although ruling out a complete role for Ca^2+^ signaling in facilitating this response may be premature. Chloride channels were not investigated in this study but many of them (CFTR, glycine‐gated and CLICs) have been indicated as regulators of macrophage and other immune cell function via phagosomal acidification, cytokine production, Ca^2+^ influx, and superoxide production. (Ikejima et al. 1997; Wheeler and Thurman 1999; Wheeler et al. 2000; Di et al. 2006; Jiang et al. 2012) The results of this study clearly indicate that exogenous ES induces a response characterized by hemocyte aggregation and collagen deposition that closely resembles a documented crustacean wound response and that *K*_V_ channels are a critical component of this response (Fontaine and Lightner 1973; Fontaine 1975).

## Conflict of Interest

None declared.
